# Acknowledgment of Reviewers

**DOI:** 10.2188/jea.JE2008AR1

**Published:** 2008-12-17

The following scientists assisted the journal by reviewing manuscripts that were submitted for consideration during the period January 1, 2008 to October 31, 2008. We gratefully acknowledge their critical evaluation and assistance in the selection of papers for publication.

**Table:** 

Adachi, Hisashi	Kurume University
Aida, Jun	Tohoku University
Akiba, Suminori	Kagoshima University
Arao, Takashi	Waseda University
Asao, Keiko	Louisiana State University
Babazono, Akira	Kyushu University
Doi, Yasufumi	Kyushu University
Fujino, Yoshihisa	University of Occupational and Environmental Health, Japan
Fujita, Toshiharu	The Institute of Statistical Mathematics
Fujiwara, Yoshinori	Tokyo Metropolitan Institute of Gerontology
Fukuda, Yoshiharu	Yamaguchi University
Fukunaga, Ichiro	Institute of Health Planning
Goto, Aya	Fukushima Medical University
Haga, Hiroshi	J.F. Oberlin University
Hamashima, Chisato	National Cancer Center
Harada, Akiko	Tokyo University
Hayashi, Kunihiko	Gunma University
Hirota, Yoshio	Osaka City University
Honda, Yasushi	Tsukuba University
Honjo, Kaori	Osaka University
Honjo, Satoshi	Fukuoka Higashi Medical Center
Hoshuyama, Tsutomu	University of Occupational and Environmental Health, Japan
Hozawa, Atsushi	Tohoku University
Iki, Masayuki	Kinki University
Inaba, Yutaka	Juntendo University School of Medicine
Ishizaki, Tatsuro	Tokyo Metropolitan Institute of Gerontology
Kadotani, Hiroshi	Kyoto University
Kadowaki, Takashi	Shiga University of Medical Science
Kamakura, Mitsuhiro	Keio University
Kaneita, Yoshitaka	Nihon University
Kasagi, Fumiyoshi	Radiation Effects Research Foundation
Kato, Noriko	National Institute of Public Health
Kawabata, Masato	Kobe University
Kawakami, Norito	Tokyo University
Kayaba, Kazunori	Saitama Prefectural University
Kikuchi, Shogo	Aichi Medical University
Kitamura, Akihiko	Osaka Medical Center for Health Science and Promotion
Kobashi, Gen	Radiation Effects Research Foundation
Kobayashi, Fumio	Aichi Medical University
Kojima, Masayo	Nagoya City University
Kokubo, Yoshihiro	National Cardiovascular Center
Kono, Suminori	Kyushu University
Koyama, Hiroshi	Gunma University
Kuboki, Makoto	Kawasaki Medical University
Kumagai, Shuzo	Kyushu University
Kuriyama, Shinichi	Tohoku University
Lee, Jung Su	Tokyo University
Lin, Yingsong	Aichi Medical University
Mafune, Kosuke	University of Occupational and Environmental Health, Japan
Marugame, Tomomi	National Cancer Center
Matsuda, Shinya	University of Occupational and Environmental Health, Japan
Matsui, Kenji	Tokyo University
Matsumura, Yasuhiro	National Institute of Health and Nutrition
Matsuo, Keitaro	Aichi Cancer Center Research Institute
Matsuyama, Yutaka	Tokyo University
Minami, Yuko	Tohoku University
Mitobe, Jiro	Infectious Disease Surveillance Center
Miyake, Yoshihiro	Fukuoka University
Mori, Mitsuru	Sapporo Medical University
Mori, Tohru	Infectious Disease Surveillance Center
Morita, Akemi	National Institute of Health and Nutrition
Morita, Manabu	Okayama University
Murakami, Hitoshi	International Medical Center of Japan
Nagai, Masaki	Saitama Medical University
Nakamura, Kazutoshi	Niigata University
Nakamura, Koushi	Kanazawa Medical University
Nakamura, Mieko	Panasonic Electric Works Co., Ltd.
Nakamura, Yasuyuki	Kyoto Women’s University
Nakaya, Naoki	Danish Cancer Society
Niino, Naoakira	J.F. Oberlin University
Nishino, Yoshikazu	Miyagi Cancer Center
Nitta, Hiroshi	National Institute for Environmental Studies
Odagiri, Yuko	Tokyo Medical University
Ohira, Tetsuya	Osaka University
Ohnishi, Hirofumi	Sapporo Medical University
Ohshige, Kenji	Yokohama City University
Ohta, Atsuhiko	Fujita Health University
Ohyama, Takaaki	Infectious Disease Surveillance Center
Oida, Yukio	Chukyo University
Ojima, Toshiyuki	Hamamatsu University School of Medicine
Okamoto, Naoyuki	Kanagawa Cancer Center
Okayama, Akira	Japan Anti-Tuberculosis Association
Omori, Takashi	Kyoto University
Ooki, Syuichi	Ishikawa Prefectural Nursing University
Osawa, Masaki	Iwate Medical University
Ozasa, Kotaro	Kyoto Prefectural University of Medicine
Sairenchi, Toshimi	Dokkyo Medical University
Saito, Isao	Ehime University
Saito, Shigeyuki	Sapporo Medical University
Saito, Tomohiro	Natonal Research Institute for Child Health and Development
Sasaki, Hideo	Hiroshima Atomic Bomb Casualty Counsil
Sasazuki, Shizuka	National Cancer Center
Sato Shin-ichi	Chiba Prefectural Institute of Public Health
Satoh, Toshihiko	Kitasato University
Sekikawa, Akira	University of Pittsburgh
Shibata, Yoshisada	Nagasaki University
Shibazaki, Satomi	Saitama Medical University
Shimazu, Taichi	National Cancer Center
Shinbo, Takuro	International Medical Center of Japan
Soda, Midori	Radiation Effects Research Foundation
Sone, Hirohito	Ochanomizu University
Suganuma, Narufumi	Kochi University
Sugimori, Hiroki	Daito Bunka University
Suzuki, Sadao	Nagoya City University
Takahashi, Hideto	Tsukuba University
Takahashi, Kunihiko	National Institute of Public Health
Takebayashi, Tohru	Keio University
Takezaki, Toshiro	Kagoshima University
Tanaka, Hideo	Aichi Cancer Center
Tanaka, Junko	Hiroshima University
Tanaka, Keitaro	Saga University
Tatemichi, Masayuki	Showa University
Tokunaga, Shoji	Kyushu University
Tsuda, Toshihide	Okayama University
Tsutsumi, Akizumi	University of Occupational and Environmental Health, Japan
Uehara, Ritei	Jichi Medical University
Wagatsuma, Yukiko	Tsukuba University
Wakai, Kenji	Nagoya University
Washio, Masakazu	St. Mary’s College
Watanabe, Shuichiro	J.F. Oberlin University
Yamagata, Zentaro	Yamanashi University
Yamaguchi, Takuhiro	Tokyo University
Yamamoto, Masaharu	Niigata University
Yamamoto, Seiichiro	National Cancer Center
Yamazaki, Shin	Kyoto University
Yatsuya, Hiroshi	Nagoya University
Yokoyama, Tetsuji	National Institute of Public Health
Yoshiike, Nobuo	Aomori University of Health and Welfare
Zamani, Saman	Kyoto University

